# Supplementary material for: Unveiling the Bioactive Potential of the Invasive Jellyfish Phyllorhiza punctata Through Integrative Transcriptomic and Proteomic Analyses
Source: Biomolecules. 2025 Aug 4;15(8):1121. doi: 10.3390/biom15081121 (PMC12383608; doi:10.3390/biom15081121)
Supplement: Supplementary file 1 [file biomolecules-15-01121-s001.zip › Suplementary Material/FileS3/PEPFOLD4/XP_065066412_13338/clusters.html]

 PEP-FOLD clusters


Download archive of all models  here   

|  |  |  |
| --- | --- | --- |
| # | file | sOPEP |
| # cluster 1 - Size: 1 |  |  |
|  | XP\_065066412\_13338-model1 | -30.0267 |
| # cluster 2 - Size: 1 |  |  |
|  | XP\_065066412\_13338-model2 | -28.2077 |
| # cluster 3 - Size: 2 |  |  |
|  | XP\_065066412\_13338-model3 | -25.3102 |
|  | XP\_065066412\_13338-model3.1 | -24.49 |
| # cluster 4 - Size: 1 |  |  |
|  | XP\_065066412\_13338-model4 | -25.2635 |
| # cluster 5 - Size: 1 |  |  |
|  | XP\_065066412\_13338-model5 | -25.0283 |
| # cluster 6 - Size: 3 |  |  |
|  | XP\_065066412\_13338-model6 | -25.0146 |
|  | XP\_065066412\_13338-model6.1 | -21.6934 |
|  | XP\_065066412\_13338-model6.2 | -20.3911 |
| # cluster 7 - Size: 1 |  |  |
|  | XP\_065066412\_13338-model7 | -24.9612 |
| # cluster 8 - Size: 1 |  |  |
|  | XP\_065066412\_13338-model8 | -22.2626 |
| # cluster 9 - Size: 2 |  |  |
|  | XP\_065066412\_13338-model9 | -21.9142 |
|  | XP\_065066412\_13338-model9.1 | -21.1279 |
| # cluster 10 - Size: 1 |  |  |
|  | XP\_065066412\_13338-model10 | -21.0982 |
| # cluster 11 - Size: 7 |  |  |
|  | XP\_065066412\_13338-model11 | -20.316 |
|  | XP\_065066412\_13338-model11.1 | -19.5481 |
|  | XP\_065066412\_13338-model11.2 | -19.4797 |
|  | XP\_065066412\_13338-model11.3 | -18.9632 |
|  | XP\_065066412\_13338-model11.4 | -18.8817 |
|  | XP\_065066412\_13338-model11.5 | -18.1181 |
|  | XP\_065066412\_13338-model11.6 | -18.0876 |
| # cluster 12 - Size: 1 |  |  |
|  | XP\_065066412\_13338-model12 | -20.0937 |
| # cluster 13 - Size: 6 |  |  |
|  | XP\_065066412\_13338-model13 | -19.9521 |
|  | XP\_065066412\_13338-model13.1 | -19.3905 |
|  | XP\_065066412\_13338-model13.2 | -18.7809 |
|  | XP\_065066412\_13338-model13.3 | -18.5698 |
|  | XP\_065066412\_13338-model13.4 | -18.4682 |
|  | XP\_065066412\_13338-model13.5 | -18.0994 |
| # cluster 14 - Size: 2 |  |  |
|  | XP\_065066412\_13338-model14 | -19.3626 |
|  | XP\_065066412\_13338-model14.1 | -17.6414 |
| # cluster 15 - Size: 2 |  |  |
|  | XP\_065066412\_13338-model15 | -19.1864 |
|  | XP\_065066412\_13338-model15.1 | -17.8315 |
| # cluster 16 - Size: 16 |  |  |
|  | XP\_065066412\_13338-model16 | -18.944 |
|  | XP\_065066412\_13338-model16.1 | -18.9029 |
|  | XP\_065066412\_13338-model16.2 | -18.8774 |
|  | XP\_065066412\_13338-model16.3 | -18.5557 |
|  | XP\_065066412\_13338-model16.4 | -18.5529 |
|  | XP\_065066412\_13338-model16.5 | -18.492 |
|  | XP\_065066412\_13338-model16.6 | -18.3487 |
|  | XP\_065066412\_13338-model16.7 | -18.3005 |
|  | XP\_065066412\_13338-model16.8 | -18.2976 |
|  | XP\_065066412\_13338-model16.9 | -18.2662 |
|  | XP\_065066412\_13338-model16.10 | -18.135 |
|  | XP\_065066412\_13338-model16.11 | -17.9838 |
|  | XP\_065066412\_13338-model16.12 | -17.9699 |
|  | XP\_065066412\_13338-model16.13 | -17.9116 |
|  | XP\_065066412\_13338-model16.14 | -17.4801 |
|  | XP\_065066412\_13338-model16.15 | -17.093 |
| # cluster 17 - Size: 5 |  |  |
|  | XP\_065066412\_13338-model17 | -18.8635 |
|  | XP\_065066412\_13338-model17.1 | -18.1661 |
|  | XP\_065066412\_13338-model17.2 | -17.9644 |
|  | XP\_065066412\_13338-model17.3 | -17.7515 |
|  | XP\_065066412\_13338-model17.4 | -16.9515 |
| # cluster 18 - Size: 1 |  |  |
|  | XP\_065066412\_13338-model18 | -18.8314 |
| # cluster 19 - Size: 4 |  |  |
|  | XP\_065066412\_13338-model19 | -18.7125 |
|  | XP\_065066412\_13338-model19.1 | -18.1773 |
|  | XP\_065066412\_13338-model19.2 | -18.124 |
|  | XP\_065066412\_13338-model19.3 | -17.9998 |
| # cluster 20 - Size: 1 |  |  |
|  | XP\_065066412\_13338-model20 | -18.6076 |
| # cluster 21 - Size: 5 |  |  |
|  | XP\_065066412\_13338-model21 | -18.5531 |
|  | XP\_065066412\_13338-model21.1 | -18.3909 |
|  | XP\_065066412\_13338-model21.2 | -18.1634 |
|  | XP\_065066412\_13338-model21.3 | -17.7055 |
|  | XP\_065066412\_13338-model21.4 | -17.6774 |
| # cluster 22 - Size: 2 |  |  |
|  | XP\_065066412\_13338-model22 | -18.5422 |
|  | XP\_065066412\_13338-model22.1 | -18.4351 |
| # cluster 23 - Size: 14 |  |  |
|  | XP\_065066412\_13338-model23 | -18.4857 |
|  | XP\_065066412\_13338-model23.1 | -18.435 |
|  | XP\_065066412\_13338-model23.2 | -18.3516 |
|  | XP\_065066412\_13338-model23.3 | -18.1817 |
|  | XP\_065066412\_13338-model23.4 | -18.1805 |
|  | XP\_065066412\_13338-model23.5 | -18.1705 |
|  | XP\_065066412\_13338-model23.6 | -18.0729 |
|  | XP\_065066412\_13338-model23.7 | -18.0077 |
|  | XP\_065066412\_13338-model23.8 | -17.9408 |
|  | XP\_065066412\_13338-model23.9 | -17.8866 |
|  | XP\_065066412\_13338-model23.10 | -17.8206 |
|  | XP\_065066412\_13338-model23.11 | -17.6542 |
|  | XP\_065066412\_13338-model23.12 | -17.505 |
|  | XP\_065066412\_13338-model23.13 | -17.2905 |
| # cluster 24 - Size: 1 |  |  |
|  | XP\_065066412\_13338-model24 | -18.4625 |
| # cluster 25 - Size: 3 |  |  |
|  | XP\_065066412\_13338-model25 | -18.3623 |
|  | XP\_065066412\_13338-model25.1 | -17.6577 |
|  | XP\_065066412\_13338-model25.2 | -17.2902 |
| # cluster 26 - Size: 3 |  |  |
|  | XP\_065066412\_13338-model26 | -18.3592 |
|  | XP\_065066412\_13338-model26.1 | -18.0108 |
|  | XP\_065066412\_13338-model26.2 | -17.6915 |
| # cluster 27 - Size: 4 |  |  |
|  | XP\_065066412\_13338-model27 | -18.1936 |
|  | XP\_065066412\_13338-model27.1 | -17.8918 |
|  | XP\_065066412\_13338-model27.2 | -17.6176 |
|  | XP\_065066412\_13338-model27.3 | -17.2524 |
| # cluster 28 - Size: 1 |  |  |
|  | XP\_065066412\_13338-model28 | -18.1921 |
| # cluster 29 - Size: 2 |  |  |
|  | XP\_065066412\_13338-model29 | -18.0363 |
|  | XP\_065066412\_13338-model29.1 | -17.6203 |
| # cluster 30 - Size: 2 |  |  |
|  | XP\_065066412\_13338-model30 | -18.0363 |
|  | XP\_065066412\_13338-model30.1 | -17.7149 |
| # cluster 31 - Size: 1 |  |  |
|  | XP\_065066412\_13338-model31 | -18.0279 |
| # cluster 32 - Size: 1 |  |  |
|  | XP\_065066412\_13338-model32 | -17.9059 |
| # cluster 33 - Size: 1 |  |  |
|  | XP\_065066412\_13338-model33 | -17.7388 |
| # cluster 34 - Size: 1 |  |  |
|  | XP\_065066412\_13338-model34 | -17.6823 |
|  |  |  |
